# Conducting Online Expert panels: a feasibility and experimental replicability study

**DOI:** 10.1186/1471-2288-11-174

**Published:** 2011-12-23

**Authors:** Dmitry Khodyakov, Susanne Hempel, Lisa Rubenstein, Paul Shekelle, Robbie Foy, Susanne Salem-Schatz, Sean O'Neill, Margie Danz, Siddhartha Dalal

**Affiliations:** 1 The RAND Corporation, 1776 Main Street, PO Box 2138, Santa Monica, CA 90401, USA; 2 Veterans Affairs Greater Los Angeles at Sepulveda, 16111 Plummer St. (152), North Hills, CA 91343, USA; 3 Veterans Affairs Greater Los Angeles Healthcare System, 11301 Wilshire Boulevard Los Angeles, CA 90073, USA; 4 Leeds Institute of Health Sciences, University of Leeds, Leeds, LS2 9JT, UK; 5 Independent Consultant, HealthCare Quality Initiatives, Newton, MA, 02459, USA; 6 Northwestern University, Feinberg School of Medicine Arthur J. Rubloff Building 420 East Superior Street Chicago, IL 60611, USA

## Abstract

**Background:**

This paper has two goals. First, we explore the feasibility of conducting online expert panels to facilitate consensus finding among a large number of geographically distributed stakeholders. Second, we test the replicability of panel findings across four panels of different size.

**Method:**

We engaged 119 panelists in an iterative process to identify definitional features of Continuous Quality Improvement (CQI). We conducted four parallel online panels of different size through three one-week phases by using the RAND's ExpertLens process. In Phase I, participants rated potentially definitional CQI features. In Phase II, they discussed rating results online, using asynchronous, anonymous discussion boards. In Phase III, panelists re-rated Phase I features and reported on their experiences as participants.

**Results:**

66% of invited experts participated in all three phases. 62% of Phase I participants contributed to Phase II discussions and 87% of them completed Phase III. Panel disagreement, measured by the mean absolute deviation from the median (MAD-M), decreased after group feedback and discussion in 36 out of 43 judgments about CQI features. Agreement between the four panels after Phase III was fair (four-way kappa = 0.36); they agreed on the status of five out of eleven CQI features. Results of the post-completion survey suggest that participants were generally satisfied with the online process. Compared to participants in smaller panels, those in larger panels were more likely to agree that they had debated each others' view points.

**Conclusion:**

It is feasible to conduct online expert panels intended to facilitate consensus finding among geographically distributed participants. The online approach may be practical for engaging large and diverse groups of stakeholders around a range of health services research topics and can help conduct multiple parallel panels to test for the reproducibility of panel conclusions.

## Background

Expert panels are an established consensus-finding method in clinical and health services research [1,2]. They often use a modified Delphi structure [3], which typically consists of two question-driven phases and one discussion phase. If conducted properly, expert panels are an invaluable tool for defining agreement on controversial subjects [4,5]. Nonetheless, panels are expensive and laborious to conduct: It is necessary to identify representative sets of experts, coordinate experts' schedules, arrange meetings, distribute panel questions in advance, and recruit a skilled facilitator to lead discussions either in person or over the phone [6,7]. Panel size is also limited to ensure effective in-person discussion. These limitations are particularly relevant to arranging panels that are inclusive enough to reflect the diversity of opinion in a broad field, such as Quality Improvement (QI).

Delphi panels can be also conducted online to facilitate the process of obtaining input from participants [8,9]. Potential advantages may include the efficient use of experts' time [9]; the ability to engage more diverse and representative panelists that may include experts from other countries [8]; the absence of expenses for postage and travel [9]; the ability to make online discussions anonymous and thus reduce possible biases based on participant status or personality [10-12]; and the benefit of contributing to the elicitation process at the time convenient to panelists [9]. Potential disadvantages, however, may include lower levels of engagement and interaction among participants, caused by their relative unfamiliarity with online tools in general and a possibility of technical difficulties accessing or using an online system, which may undermine panelists' willingness to participate and affect the quality of deliberations and outputs [13].

While potentially useful, online expert panels with a discussion board functionality are a relatively new phenomenon. Previous research also identified a number of concerns about the quality of online interaction [14], including variable participation rates, information overload, and difficulties in following discussion threads [15,16]. The best panel size for online discussion is also unknown. Very large panels, for example, might cause coordination problems [12] or impede effective interaction. Very small panels, in turn, may not benefit from fruitful discussions because participants may not feel obliged to contribute to anonymous discussions [17]. In addition, we know that in-person panels given the same information may come up with different conclusions [18,19], yet we do not know the magnitude of this effect for online panels.

To evaluate both the quality and usefulness of online expert panels, it is necessary to compare them to traditional face-to-face panels. Nonetheless, before a randomized controlled trial can be conducted, a feasibility and replicability study of using online panels should be performed first. Therefore, in this article, we evaluate the feasibility of conducting online expert panels for engaging a large, diverse group of stakeholders and discuss the replicability of findings across panels of different size.

To do so, we conducted four concurrent online expert panels of various sizes that evaluated the key definitional features of the term "Continuous Quality Improvement" (CQI) and assessed panelist participation across all panel phases. We then tested levels of agreement within and between panels. We also analyzed panelists' satisfaction with the online process and specifically assessed whether it differed between panelists representing different stakeholder groups. Finally, we explored the effects of panel size on participation rates, agreement, and participants' satisfaction.

An online approach can be considered feasible if panel participation is relatively high (e.g., above a typically expected 45-50% participation rate [20]), panelists achieve consensus, and participants are generally satisfied with the process. Panel results can have an acceptable level of replicability if the level of inter-panel agreement is fair (kappa coefficient is in the .2-.4 range) or above. A finding that the online panel approach was feasible would show that the method has promise not only for advancing appropriate terminology use in QI, but also for facilitating decision-making in other fields of health services research. Moreover, it would also indicate that a study comparing the results of a face-to-face and an online Delphi-like panel should be conducted.

## Method

To explore the feasibility of an online approach and to evaluate the replicability of panel findings, we convened and asked 4 online panels to define the appropriate use of the term "Continuous Quality Improvement"^1^. The QI field is rapidly developing [21]. Healthcare organizations are increasingly investing in QI approaches, and funders and journals support a growing level of QI research. Major communication challenges have arisen, however, due to lack of consensus around QI terminology use [22]. For example, two studies may both report the use of "CQI" but define or operationalize it so differently that they might as well report entirely different interventions [23]. Achieving improved communication thus requires consensus around key terms and must engage the perspectives of both QI practitioners and more research-oriented stakeholders. In this study, we used online expert panel methods to attempt to engage both stakeholder types.

LR and SSS used their professional networks to invite Institute for Healthcare Improvement faculty, members of the editorial boards from leading QI research journals, evaluators of Robert Wood Johnson Foundation (RWJF) quality programs, and RAND patient safety and QI experts to participate in this study. Participants were asked to nominate other QI professionals and health services researchers. Out of 259 professionals contacted, 119 agreed to participate.

As part of the agreement to participate, we asked participants to self-identify themselves as primarily practitioners, primarily researchers, or both equally. We used stratified random sampling to assign participants to one of two small (n1 = 19, n2 = 21) or two large (n3 = 40, n4 = 39) panels and balance panels with regard to the number of researchers and practitioners. Participants were not informed about the size of their panels or the total number of panels. While participants knew that the study would consist of three phases, consistent with the RAND/UCLA Appropriateness Method manual [3], we did not explicitly instruct panelists to develop consensus. The study was determined to be exempt from the IRB review by the RAND's Human Subjects' Protection Committee.

ExpertLens is one system for conducting online expert panels. It was created by an interdisciplinary team of researchers at the RAND Corporation [24]. It uses a modified-Delphi elicitation structure and replaces traditional face-to-face meetings with asynchronous, unmoderated online discussion boards. The online process used in this study consisted of three phases; each phase was limited to one week. In Phase I, panelists rated 11 features of CQI initiatives on four dimensions, including the importance of a feature for a definition of CQI. The initial features came from earlier consensus work that used a traditional expert panel process [23], but study participants could also add other important features they felt were missing. In Phase II, panelists saw their own responses as well as the medians and quartiles of their panel responses to Phase I questions. They also participated in asynchronous, anonymous, and unmoderated online discussions with the same group of colleagues in each panel. Phase II was the feedback phase that allowed panelists to review the panel response by looking at measures of central tendency and dispersion and discuss their ideas anonymously, without being influenced by the status of other panelists [12]. In Phase III, panelists re-answered Phase I questions. In the optional post-completion survey, participants rated additional features mentioned in Phase I and answered questions about their experiences participating in the online expert panel.

In line with consensus methods guidelines, the definitions of importance of a particular CQI feature, as well as of the level of consensus, were determined in advance [4]. We considered a feature to be important for a CQI initiative if a panelist rated it as > 3 on a 5-point importance scale. We also used an a priori definition of consensus. If more than two-thirds (> 66.6%) of panelists agreed on the importance of a particular feature, we argued that consensus was achieved [25]. We used mean absolute deviation from the median (MAD-M) as a measure of disagreement within panels and treated a reduction in its values between phases as a sign of increased consensus [3,26]. MAD-M is the preferred measure of disagreement in expert panels that has been widely used since 1980s when the RAND/UCLA Appropriateness Method was originally created. It is a good measure of disagreement because it is not affected by extreme observations and measures deviation from the median, a measure of central tendency typically used in consensus development and in this study [26]. Finally, we used four-way kappa to assess agreement between panels, treating the data as ordinal and using a weight matrix comprising the squared deviations between scores [27].

## Results

### Participation

Out of 119 individuals who expressed interest in participating in the ExpertLens process, 77% completed Phase I (Table 1). Participation rates varied from 63% in a small panel to 83% in a large panel. In total, 62% of Phase I participants contributed to Phase II discussions. 66% of those invited to the study, and 87% of Phase I participants, also participated in Phase III. There was no statistically significant difference in participation levels for Phase I and III between the panels.

**Table 1:** Participation in All Phases of the Study

*Participation Characteristics*	*Panel A*	*Panel B*	*Panel C*	*Panel D*	*Total*
**Total number of invited participants**	21	19	40	39	119

***Phase I***					

**Number of Phase I participants**	15	12	33	31	91
**Participation rate in Phase I**	71%	63%	83%	80%	77%

***Phase II***					

**Number of participants posting comments in Phase II**	9	6	25	16	56
**% of active discussion participants**	60%	50%	76%	52%	60%
**Total number of discussion threads**	7	6	16	11	10
**Average number of threads initiated per participant**	.77	1	.64	.68	.77
**Total number of discussion comments**	18	21	89	45	43
**Average number of comments per participant**	2	3.5	3.6	2.8	3
**Range of number of comments per participant**	1-5	1-6	1-9	1-9	1-9

***Phase III***					

**# of Phase III participants**	10	10	32	27	79
**Participation rate in Phase III**	67%	83%	97%	87%	87%
**Participation rate in all phases**	48%	53%	80%	69%	66%

In each panel, between 50% and 76% of Phase I participants contributed to Phase II discussions (Table 1). Discussion participation rates and the average number of comments per participant did not vary significantly across the panels in relationship to panel size. One of the large panels (Panel C) had the most active discussion, with 76% of panel members participating by posting 16 discussion threads with 89 comments (On average, each Panel C participant initiated .64 discussion threads and made 3.56 comments). Table 2 illustrates the type of discussion the groups carried out by showing Panel C's discussion of Feature 5 "Use of evidence"-- one of the eleven potential CQI features the panelists assessed.

**Table 2:** A Sample Discussion Thread: Feature 5 "Use of Evidence"

*Participant ID*	*Discussion Comments*
62	This score was most surprising to me. I think many improvement efforts - particularly those undertaken by learners - fail to adequately use the evidence. This is also the link between evidence-based practice (or evidence-based medicine) and QI. When evidence is weak for a change or if the focus of the change is more administrative, outcomes suffer. Strong evidence for a change should be a key element in any improvement effort.
58	I rated this as less important in the definition of QI...because, while I think using evidence relevant to the problem is important when strong evidence exists, I also think there are cases where evidence is lacking, but improvement still needs to happen. Therefore, I didn't think it could be a critical feature of the definition of QI, mostly because of the 2nd case I mentioned.
78	I agree with this last comment and rated this feature low for the same reasons.
51	Agree with 58 and 78
60	Agree with 58, 78, 51. Furthermore, one key reason for the "rapid cycle" element is the fact that prior evidence may not exist, or may not be relevant. The best evidence for the change is whether it is effective in the current context. Prior evidence, if available, should be consulted, but (a) it's not always available, and (b) even if available is not always relevant.
42	Targeting solutions to problems may help generate evidence that a given intervention is effective. (See The Joint Commission's Targeted Solutions Tool, which allows organizations to find the problem(s) they have and they pick the corresponding solution (starting with hand hygiene).
67	Agree with 62 on the assumption that, in the absence of scientific evidence, expert judgment is the next best thing and would constitute the available "evidence" - as is the case with much of what is asked about this process.

### Consensus

Although participants were not instructed to reach consensus, all panels were able to do so on four out of eleven features in Phase I; three panels agreed on three additional features, and two panels on one further feature (Table 3). Three features were not judged as important in any panel. In Phase III, after group feedback and discussion, all panels agreed on the importance of only three of the four features identified in Phase I; three panels agreed on five other features (Table 3). Of the features that were not judged as important by any panel in Phase I, one feature (#5) was then deemed important by two panels, following Phase II feedback and discussion. Table 2 illustrates comments made about this feature in Panel C. While some differences in opinion about the importance of Feature 5 still exist in Panel C, participants agreed that this feature is important to the definition of CQI in Phase III. Two features, however, were still not deemed important by any panel.

**Table 3:** Feature Importance to the Definition of a CQI Initiative and Agreement between Panels

*Features*	*Panel A*, *Phase I*	*Panel B*, *Phase I*	*Panel C*, *Phase I*	*Panel D*, *Phase I*	*Total, * *Phase I*	*Panel A*, *Phase III*	*Panel B*, *Phase III*	*Panel C*, *Phase III*	*Panel D*, *Phase III*	*Total,* *Phase III*
***Feature 1***	**14** **93.3%** **.53**	**12** **100% ** **.25**	**30** **90.9%** **.39**	**30** **96.8%** **.45**	**86** **94.5%** **.42**	**10** **100%** **.3**	**10** **100%** **.2**	**32** **97%** **.24**	**26** **100%** **.27**	**78** **98.7%** ** .25**
***Feature 2***	**13** **92.9%** **.57**	6 50% .92	**28** **82.4%** **.71**	**21** **67.7%** **.87**	**68** **74.7%** **.77**	**9** **90%** **.6**	5 50% .8	**28** **84.8%** **.64**	**18** **69.2%** **.89**	**60** **75.9%** **.69**
***Feature 3***	8 57.1% 1	5 41.7% .92	20 58.8% 1.03	16 51.6% .97	49 53.8% 1.01	5 50% .8	1 10% .3	19 59.4% .91	16 59.3% .85	41 51.9% .94
***Feature 4***	**13** **92.9% ** **.5**	6 50% 1.17	**25** **3.5%** **7 .91**	**26** **74.2%** **.94**	**67** **73.6%** **.97**	**8** **80%** **.6**	6 60% .6	**28** **84.8%** **.73**	**24** **88.9%** **.63**	**66** **82.5%** **.75**
***Feature 5***	6 42.9% 1.14	7 58.3% 1.08	18 54.5% 1	19 61.3% .68	50 55.6% .94	5 50% .8	**7** **70%** **.8**	**23** **71.9%** **.69**	15 55.6% .59	50 63.3% .68
***Feature 6***	**11** **84.6%** **1.21**	**9** **75%** **.75**	**30** **88.2%** **.62**	**27** **87.1%** **.74**	**77** **85.6%** **.77**	**9** **90%** **.5**	**9** **90%** **.4**	**29** **90.6%** **.47**	**24** **88.9%** **.52**	**71** **89.9%** ** .51**
***Feature 7***	**11** **78.6%** **1**	**9** **75%** **.58**	**27** **79.4%** **.68**	**28** **90.3%** **.55**	**75** **82.4%** **.67**	**8** **80%** **.9**	**9** **90%** **.5**	**25** **75.8%** **.73**	**26** **96.3%** **.52**	**68** **85%** **.65**
***Feature 8***	6 46.2% 1.15	**8** **66.7%** **.92**	20 58.8%t 1.03	**22** **71%** **.77**	56 62.2% .96	**6** **66.7%** **.67**	**7** **70%** ** .7**	21 63.6% .76	**18** **66.7%** **.85**	52 65.8% .77
***Feature 9***	**11** **84.6%** **.46**	**10** **83.3%** **.58**	N/A	**28** **90.3%** **.48**	**49** **87.5%** **.58**	**10** **100%** **.1**	**10** **100%** **.4**	18 62.1% .79	**26** **96.3%** **.37**	**64** **84.2%** **.73**
***Feature 10***	9 64.3% 1.07	6 50% .92	14 41.2% 1.38	16 51.6% 1.23	45 49.5% 1.27	4 40% 1.2	3 30% .6	9 28.1% 1.06	13 48.1% 1.48	29 36.7% 1.05
***Feature 11***	**13** **92.9%** **.71**	**8** **66.7%** **1.17**	**22** **75.9%** **.83**	**23** **74.2%** **.84**	**66** **76.7%** **.92**	**10 ** **100%** **.5**	6 60% 1	**26** **81.3%** **.59**	**19** **70.4%** **.78**	**61** **77.2%** **.82**

(Frequencies, % of responses higher than 3 on a 1-5 importance scale, MAD-M)

Question: How important is this feature to the definition of a CQI initiative? Response scale: 1 = Not Important - 5 = Very Important

Cells with bold font indicate panels where the majority (> 66.6%) of participants think that this feature is important for the definition of CQI.

**Figure 1:**
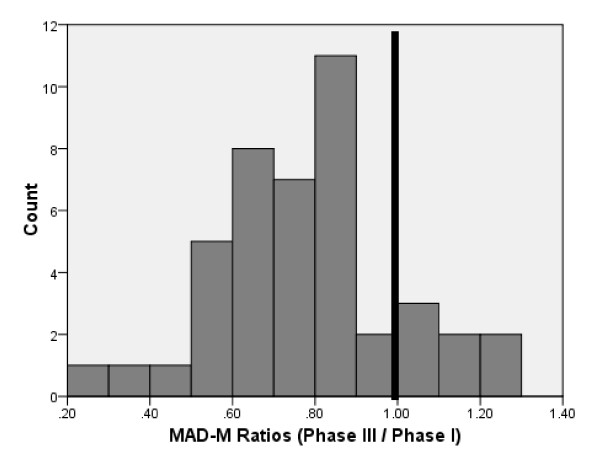
**Distribution of Phase III/Phase I MAD-M Ratios**. Figure 1 graphically depicts the ratio of MAD-M values in Phase III relative to Phase I; a value below 1.0 illustrates decrease in disagreement.

The MAD-M values for features where consensus was reached ranged from .25 to 1.21 in Phase I and from .1 to .89 in Phase III. In 36 out of 43 cases^2 ^(84%), the MAD-M values decreased between Phase I and Phase III. Figure 1 graphically depicts the ratio of MAD-M values in Phase III relative to Phase I; a value below 1.0 illustrates decrease in disagreement. Results suggest that panelists' answers clustered more around the group median after statistical feedback and discussion, meaning that agreement among panelists increased between Phase I and Phase III.

### Replication

By design, we used stratified random sampling and identical elicitation procedures to test for reproducibility of panel conclusions. Our Phase III results show some variation between panels (See Table 3). For instance, in Panel D, eight features were rated as important for the definition of CQI. For Panels A and C, however, the definition of CQI consisted of seven features; yet not all of them were the same. Finally, for Panel B, the CQI definition consisted of only six features.

The four-way kappa, which measures the level of agreement between the four panels, was equal to .36 and thus fell within the .20-.40 range that typically illustrates fair agreement [28,29]. Agreement between two larger panels was slightly higher (pairwise kappa = .38) than that between two smaller panels (pairwise kappa = .24). Panels A and D, however, had a 100% agreement in Phase III.

Nonetheless, Table 3 shows that all four panels agreed on the status of five out of eleven CQI features by uniformly considering them either important or not important. Five other features were endorsed as important by three panels; and one additional feature was endorsed by two panels. Therefore, this finding supports the stance that three features endorsed by all four panels should be considered important to the definition of CQI, two features that were not rated as important by any of the panels should not be discussed further, and five features endorsed by three panels require additional discussions.

### Satisfaction

While there was some variation, participants were generally satisfied with the ExpertLens process (Table 4). All satisfaction questions had 7-point response scales, where 1 = Strongly Disagree, 2 = Disagree, 3 = Slightly Disagree, 4 = Neutral, 5 = Slightly Agree, 6 = Agree, and 7 = Strongly Agree. The mean values were rounded to the nearest whole number. Although panelists agreed slightly that participation in the exercise was interesting (mean = 5.31, sd = 1.32) and the survey instrument was easy to use (mean = 4.78, sd = 1.40), they had a neutral opinion on whether participation in this exercise was frustrating (mean = 3.57, sd = 1.80). CQI practitioners were significantly less likely to think that the instrument was easy to use, compared to researchers or those self-characterized as both (p = .025).

**Table 4:** Results of the Post-Completion Survey (N = 76)

*Statement*	*Researchers*	*Researchers and Practitioners*	*Practitioners*	*Large Panels*	*Small Panels *	*Total*
***1. Participation in the exercise was interesting***	5.68 (1.08)	4.96 (5.18)	5.18 (1.01)	5.29 (1.36)	5.39 (1.24)	5.31 (1.32)
***2. The survey instrument was easy to use***	5.42 (1.26)	5.03 (1.76)	4.11 (1.81)*	5.05 (1.52)	4.72 (2.05)	4.78 (1.40)
***3. Participation in this exercise was frustrating***	3.34 (1.58)	3.69 (2.24)	3.78 (1.48)	3.77 (1.82)	2.95 (1.61)†	3.57 (1.80)
***4. I was comfortable expressing my views in the discussions***	5.35 (1.33)	5.81 (1.11)	5.33 (1.19)	5.43 (1.29)	5.78 (1.00)	5.51 (1.23)
***5. The exercise brought out views I hadn't considered***	5.06 (1.03)	4.52 (1.87)	4.61 (1.50)	4.75 (1.54)	4.79 (1.36)	4.76 (1.49)
***6. The discussions gave me a better understanding of the issues***	4.75 (1.37)	4.35 (1.70)	4.72 (1.53)	4.51 (1.59)	4.89 (1.24)	4.61 (1.51)
***7. Group members debated each others' viewpoints during the discussions*.**	4.66 (1.10)	4.04 (1.68)	4.78 (1.40)	4.74 (1.26)	3.63 (1.54)*	4.47 (1.41)
***8. The discussion in Phase II caused me to revise my original responses***	4.13 (1.45)	4.04 (1.72)	4.61 (1.46)	4.10 (1.53)	4.56 (1.62)	4.21 (1.55)
***9. I had trouble following discussions***	3.75 (1.67)	3.62 (1.86)	4.39 (1.42)	4.03 (1.71)	3.28 (1.53)†	3.86 (1.69)
***10. I will participate in another ExpertLens process on minimum quality standards for CQI reporting***	5.75 (1.01)	4.44 (2.10)	4.88 (1.65)**	5.02 (1.62)	5.32 (1.95)	5.09 (1.70)

(Means and standard deviations)

The first 9 statements were rated on a 7-point agreement scale, where 1 = Strongly Disagree, 4 = Neutral, and 7 = Strongly Agree.

The last statement ware rated on a 7-point likelihood scale, where 1 = Very unlikely and 7 = Very likely.

†p ≤ .1, * p ≤ .05; ** p ≤ .01; *** p ≤ .001

Significance levels presented in the third column of the table refer to the differences in opinions between researchers, researchers and practitioners, and practitioners; significance levels presented in the sixth column refer to the differences in opinions between large and small panels.

Participants expressed generally positive opinions about the Phase II online discussion and the value it brought to the online expert elicitation process. Panelists agreed that they were comfortable expressing their views in the discussions (mean = 5.51; sd = 1.23). They also agreed slightly that the exercise brought out the opinions they had not considered (mean = 4.76; sd = 1.49) and that discussions gave them a better understanding of issues (mean = 4.61; sd = 1.51). Finally, panelists' opinions were close to neutral on whether panel members debated each others' viewpoints (mean = 4.47; sd = 1.41), whether discussions caused them to revise their original responses (mean = 4.21; sd = 1.55), and whether they had trouble following discussions (mean = 3.86; sd = 1.69).

While satisfaction with the online process and discussions varied slightly between the panels, there typically was no statistically significant panel size effect. The only exception was that panelists in larger panels were significantly more likely than those in smaller panels to agree that participants debated each others' viewpoints during discussions (mean = 4.74, sd = 1.26 vs. mean = 3.63, sd = 1.54; p = .002).

Finally, participants said that they would likely participate in a similar online panel in the future (mean = 5.09; sd = 1.70); researchers, however, were significantly more likely than the other two groups of panelists to express their willingness to participate (p = .009).

## Discussion

The study was designed to explore the feasibility of conducting online expert panels and to examine experimental replicability of their findings. We focused specifically on the issues of expert participation, consensus development, agreement across panels, and participant experiences. We also investigated the effects of the panel size on participation rates and satisfaction with the ExpertLens process used to conduct online panels. Our exploratory study shows that online expert panels may be a practical approach to engaging large and diverse groups of stakeholders in finding consensus on key language issues within an evolving field, such as QI. It also supports the results of previous research showing that virtual panels may potentially expedite the elicitation process, minimize burden on participants, allow the conduct of larger and more diverse panels, and include geographically distributed participants [8,9].

Overall, CQI stakeholders demonstrated strong commitment to improving CQI language, and the study participation rate was high, with 66% of participants, who did not receive any honoraria, engaging in all phases of the online elicitation. This number compares favorably to both the 45-50% typically expected participation rate in a traditional Delphi study [20] and the 49% participation rate in a recent online Delphi with just two questions phases [8].

Moreover, our panelists generally expressed positive attitudes towards an online approach, finding the elicitation process interesting, the online system easy to use, and the discussion component helpful for improving their understanding of the issues and clarifying their positions. Typical average satisfaction scores were equal to, or above, "agreed slightly" on positively worded satisfaction items.

Although participation levels did not vary significantly across the panels of different size, the perception of a two-way information exchange, as measured by the post-completion survey questions, was significantly higher in larger than in smaller panels. Therefore, the number of invited participants in online consensus panels may need to be higher than in traditional panels to ensure that the critical mass of participants is achieved not only during the questions but also during the discussion phases [30]. On the one hand, inviting a larger number of panelists may increase the panel's representativeness [12] and allow for exploring the differences not only between, but also within stakeholder groups. On the other hand, our largest panel (n = 40) was still of a size we considered reasonable for engaging a high percent of panelists in the discussion; having a very large number of panelists might have a deleterious effect on discussion participation.

Finally, our study suggests that the online approach can be used to conduct multiple parallel panels to test for the reproducibility of panel conclusions. In this study, the level of agreement between panels was fair as measured by four-way kappa [28,29], and roughly a quarter of all potential features was judged important by all four panels. The comparison across panels is crucial information when evaluating the potential replicability of panel decisions and provides an indication of the degree of confidence in the robustness of decisions across panels. By the end of Phase III, all four panels agreed on the status of five out of eleven CQI features. The data feedback and discussion features of the online system appeared to reduce MAD-M values (i.e., increase the level of agreement) between Phase I and Phase III without forcing participants into consensus. By virtue of answering the same questions twice and discussing their perspectives, all four panels agreed on the importance of three out of eleven features to the definition of CQI, and on the lack of importance of two other features.

While our study illustrates the feasibility of conducting online expert panels, it, nonetheless, has some limitations. In terms of panel size, our results reflect only a modest panel size range; we did not test extremely small or large sizes. Furthermore, we do not know how well we represented QI researchers versus QI practitioners in our sample, because we only can categorize those who actually signed up to participate; however, our Phase I response rate of 77% does not suggest a high level of bias in this regard. Finally, in terms of achieved participation rates and panel results, the findings may primarily reflect the dedication of CQI stakeholders and may not apply to other topics and applications. Previous studies using this online approach [13], however, also indicate that this process can help obtain input from large, diverse, and geographically dispersed groups of stakeholders who try to foster exchange and find consensus on often controversial topics and policy questions. Nonetheless, further experimental research is necessary to validate these findings.

## Conclusions

In summary, our study illustrates the feasibility of conducting online expert panels and explores the replicability of panel findings. Online panels may be helpful for engaging large and diverse groups of stakeholders for defining agreement on controversial subjects, such as refining and understanding QI language. Additional tests of ExpertLens and other online panel tools, however, should further determine their acceptability and validity as an alternative, or an addition, to a face-to-face panel process for a range of health services research topics and provide detailed information about the best ways to configure and carry out online expert panels.

## Competing interests

DK and SD are developers of the ExpertLens system. The RAND Corporation, a non-profit research institution, is the registered owner of the ExpertLens trademark.

## Authors' contributions

All authors have contributed substantially to the manuscript. DK contributed to the study design, was responsible for data collection, performed the data analysis, and wrote the first draft of the manuscript. SH contributed to the study design and data collection, advised on data analysis, and contributed to the manuscript. LR contributed to the study design and manuscript writing. SO led participant randomization, was involved in the study design and data collection processes, and commented on the manuscript. PS, RF, SSS, MD, and SS were involved in the conception and design of the study and the data analysis strategy, provided advice on data interpretation, and contributed to the revisions of the manuscript. All authors have approved the final version of the manuscript.

## Endnotes

1. This paper explores the feasibility of the online panel approach; the results on consensus on specific defining features of CQI will be reported elsewhere.

2. By case we mean a feature in each group. We asked questions about 11 features in 4 panels. In Panel C, one question was not asked in Phase I. Therefore, we had 43 cases total in Phase I.

## Pre-publication history

The pre-publication history for this paper can be accessed here:

http://www.biomedcentral.com/1471-2288/11/174/prepub
